# 

**Published:** 1915-06-05

**Authors:** 


### Gifts and Bequests.

The London Lock Hospital has received a grant of £200 from the trustees of Smith's Charity, and the late Mr. William Buchan Gilbert has bequeathed to the Female Hospital a legacy of £30.

Mr. George Priestman, chairman of the house committee of the Bradford Royal Infirmary, has given a donation of £100 to provide an *x*-ray apparatus for deep-seated therapy at the Royal Infirmary.

The late Mr. Jesse Mellor, of Bradford, who died on March 20, leaving gross estate to the value of £32,906, has left the following bequests to Bradford charities: The Royal Infirmary, £2,000; the Royal Eye and Ear Hospital, £2,000; the Children's Hospital, £2,000; St. Catherine's Home for Cancer and Incurables, £500; and Royal Institution for the Blind, £100.

The Right Hon. Edward George Augustus Harcourt, sixth Earl Mountcashell, of Wells, Somerset, who died on April 1, aged eighty-five, left £500 to Dr. Barnardo's Homes, £100 to the Wells Cottage Hospital, £1,000 to his housekeeper, £800 to his housemaid, £500 to his gardener, £350 and £300 to two other maidservants, £100 to a manservant, and one year's wages to each servant of five years' service.

The Clothworkers' Company have made the following bequests to hospitals: King Edward's Hospital Fund, £500; King's College Hospital and London Hospital, £200 each; City of London Lying-in Hospital, London School of Medicine for Women, Middlesex Hospital, Queen's Hospital for Children, Royal Dental Hospital of London, Royal National Orthopædic Hospital, and Royal Aid Society, £100 each; Royal Sea Bathing Hospital, 50 guineas; Hospital for Epilepsy and Paralysis (Maida Vale), Italian Hospital, Paddington Green Children's Hospital, Royal National Hospital for Consumption, All Saints' Convalescent Hospital, Eastbourne, £50 each; Miller General Hospital, £34; St. Andrew's Convalescent Home, Folkestone, £25; and the Convalescent Home for Poor Children, St. Leonards, £20.

### The Book Market.

### Institutional and Public Reports.

Middlesex Hospital: Report for 1914.
Gloucestershire Royal Infirmary: Report for 1914.
St. Bartholomew's Hospital, Rochester: Report for 1914.
Cancer Charity of the Middlesex Hospital: Report for 1914.
Preston Royal Infirmary: Forty-fifth Annual Report for 1914.

### A Cautionary Notice.

The Secretary of the London Hospital wishes to warn employers of nurses that Nurse Lovelock, 78 Lancaster Road, Notting Hill, who professes to have been trained at the London Hospital, was never trained there.

*To the Editor of* The Hospital.

Sir,—We should feel greatly obliged if you would kindly find space for the above in your news columns.—We are, Sir, yours faithfully,

May 31. Charles Barker and Sons, Ltd.

RATES OF SUBSCRIPTION: Twelve months, 6/6; Six months, 4/-; Three months, 2/-, post free to any address in the United Kingdom. Abroad, Twelve months, 8/8; Six months, 5/-; Three months, 2/6, post free.—Address The Subscription Dept., "THE HOSPITAL," The Hospital Building, 28/29 Southampton Street, Strand, London, W.C.

*Now Ready.* Twenty-Sixth Year of Publication. Over 1,000 pages. In Scarlet Cloth.

Price **10/6** net. By post, **10/11.**

**BURDETT'S**

**HOSPITALS and CHARITIES, 1915.**

The Year Book of Philanthropy and Hospital Annual.

**Edited by Sir HENRY BURDETT, K.C.B., K.C.V.O.**

"The Ideal of what a work of reference ought to be."—*The Times.*

**The volume for 1915 is the 26th Issue of "Hospitals and Charities," and contains SPECIALLY WRITTEN CHAPTERS on:—**

The Volume of Charity, 1913. The Future of the Hospitals. The King's Fund and the League of Mercy. The Nursing Department in 1913 and its Cost. Hospital Sunday. Hospital Saturday. Missions. Orphanages. The Blind. The Deaf and Dumb. Hospital Finance—Income, 1913; Expenditure, 1913. The United States, Canada, Australasia, and India. Convalescent Institutions. Hospital Construction, 1914.

**ALL WITH CAREFULLY ANALYSED FIGURES, AND A DIRECTORY OF**

Medical Universities, Colleges, and Schools.—London Hospitals.—London Dispensaries.—Metropolitan Asylums Board Hospitals.—Poor-Law Infirmaries in London and the Provinces.—Provincial Hospitals.—Provincial Infectious Hospitals.—Provincial Dispensaries.—Scotch Hospitals.—Scotch Dispensaries.—Irish Hospitals.—Lunacy Board and Lunatic Asylums: Indian, British, and Colonial.—Military and Naval Hospitals.—Convalescent Homes.—Sanatoria for Consumption.—Retreats for Inebriates.—Private Asylums.—Nursing Institutions.—Indian, Colonial, and American Hospitals.—Hospitals for Insane in the United States of America.—Administrative and Collective Societies.—Missionary and Religious Societies.—Orphanages, Homes, and Charities.—Institutions for the Blind.—Institutions for the Deaf and Dumb.—London County Council Centres for Blind Children.—London and Provincial Institutions for the Deaf and Dumb.—London County Council Centres for Deaf and Dumb Children.—Hospitals and Homes for Incurables.—Establishments for the Relief of Epilepsy.—Vaccine Lymph Establishments.—Reformatory and Industrial Schools.

**Obtainable of all Booksellers.** **Publishers: The Scientific Press, Ltd., London.**

